# Regulation of T-Cell Signaling by Post-Translational Modifications in Autoimmune Disease

**DOI:** 10.3390/ijms19030819

**Published:** 2018-03-12

**Authors:** Taku Kuwabara, Yukihide Matsui, Fumio Ishikawa, Motonari Kondo

**Affiliations:** Department of Molecular Immunology, Toho University School of Medicine, 5-21-16 Omori-Nishi, Ota-ku, Tokyo 143-8540, Japan; ym04083@yahoo.co.jp (Y.M.); fumioimm@toho-u.ac.jp (F.I.); motonari.kondo@med.toho-u.ac.jp (M.K.)

**Keywords:** T cell, autoimmune disease, post-translational modification

## Abstract

The adaptive immune system involves antigen-specific host defense mechanisms mediated by T and B cells. In particular, CD4^+^ T cells play a central role in the elimination of pathogens. Immunological tolerance in the thymus regulates T lymphocytes to avoid self-components, including induction of cell death in immature T cells expressing the self-reactive T-cell receptor repertoire. In the periphery, mature T cells are also regulated by tolerance, e.g., via induction of anergy or regulatory T cells. Thus, T cells strictly control intrinsic signal transduction to prevent excessive responses or self-reactions. If the inhibitory effects of T cells on these mechanisms are disrupted, T cells may incorrectly attack self-components, which can lead to autoimmune disease. The functions of T cells are supported by post-translational modifications, particularly phosphorylation, of signaling molecules, the proper regulation of which is controlled by endogenous mechanisms within the T cells themselves. In recent years, molecular targeted agents against kinases have been developed for treatment of autoimmune diseases. In this review, we discuss T-cell signal transduction in autoimmune disease and provide an overview of acetylation-mediated regulation of T-cell signaling pathways.

## 1. Introduction

T cells play an essential role in defending the body against pathogens. CD4^+^ T cells promote the elimination of infectious agents by activating other types of cells, such as macrophages, whereas CD8^+^ T cells induce cell death in virus-infected cells [1]. After the binding of the peptide-major histocompatibility complex (MHC) complex and T-cell receptor (TCR), T cells detect pathogen infection specifically based on prior exposure and maintain memory cells to combat subsequent infection. This class of cells is characterized by the ability to distinguish between self- and non-self-components. Non-self-components include exogenous antigens and endogenous antigens that show inappropriate expression, such as tumor-associated antigens. Typically, T cells do not react to self-tissues because their activities are controlled by immune tolerance mechanisms preventing such responses. Immune reactions to self-components via the TCR occur when this tolerance is attenuated. Tissue function can become disrupted if such self-directed responses become severe enough to cause inflammation. Dysfunction of tissues triggered by self-reactive T cells is termed autoimmune disease [2,3].

Stimulation of T cells with antigens or cytokines induces the phosphorylation of signaling molecules, which is a key event controlling gene expression, cell motility, and proliferation [4]. In most cases, protein phosphorylation acts as a molecular switch [5,6]. In the proximal region of the TCR complex, for example, phosphorylation of the CD3 ζ-chain by lymphocyte-specific protein tyrosine kinase (Lck) starts a signaling cascade that continues to other downstream enzymes [7]. Dephosphorylation of proteins can also activate enzymes in some cases; for example, the nuclear translocation of the transcription factor nuclear factor of activated T-cells (NFAT) is induced by the dephosphorylation of several phosphorylated serine residues in its N-terminal region by the phosphatase calcineurin [8]. The activation state of T cells is greatly influenced by regulation of protein function via phosphorylation. However, recent studies have also shown that these signaling molecules can also be regulated by acetylation [9,10,11]. That is, nuclear proteins can be modified by acetyl groups, and cytoplasmic proteins, such as receptors and signal transducers and activators of transcription (STAT) family transcription factors, can undergo acetylation by type I interferon (IFN) stimulation. Acetylation of proteins is essential for IFN signal transduction [10]. The cytokine interleukin-2 (IL-2) is essential for T-cell differentiation and function; acetylation of signaling molecules by IL-2 receptor (IL-2R) stimulation can negatively regulate T-cell function [9].

Peripheral tolerance is the result of anergy or T-cell suppression. Full activation of T cells requires recognition of an antigen by the TCR and recognition of co-stimulators, mainly CD80 and CD86, by CD28. A prolonged antigen signal alone may lead to anergy [12,13]. It is likely that self-antigens are displayed to T cells in the absence of strong co-stimulation. Anergic T cells show a block in TCR-induced signal transduction, which is attributable to decreased TCR expression and recruitment to the TCR complex of inhibitory molecules, such as tyrosine phosphatases. When T cells recognize self-antigens, they may engage inhibitory receptors of the CD28 family, such as cytotoxic T lymphocyte antige-4 (CTLA-4) and programmed death-1 (PD-1) [12,14,15], which function to terminate the T cell response. CTLA-4 competes with CD28 for CD80/CD86 co-stimulators and excludes CD28 from the site of the TCR. CTLA-4 delivers inhibitory signals that negate the signals triggered by the TCR. Another inhibitory receptor is PD-1 [12,16], which recognizes two ligands, programmed death 1 ligand 1 (PD-L1) and PD-L2, expressed on antigen-presenting cells. This ligand binding leads to suppression of T cells. Anergy and T-cell suppression mechanisms function cooperatively to prevent dangerous autoimmunity.

Dysfunction of T-cell regulatory pathways causes autoimmune disease. Both phosphorylation and acetylation are closely involved in T-cell function. In this paper, we provide an overview of T-cell signaling regulation and autoimmune disease in anticipation of a new era in which these conditions can be managed by targeting cytoplasmic acetylation. We focus on autoimmune responses observed in animal models as well as their corresponding diseases.

## 2. T-Cell Signal Transduction Pathways

T cells express many types of receptors on their surfaces. After sensing some ligands, the receptors induce gene expression or cell motility. Thus, receptors play various roles. Although T cells express many receptors, the TCR and cytokine receptors directly influence their immune reactions.

### 2.1. TCR Signal Transduction

**Figure 1:**
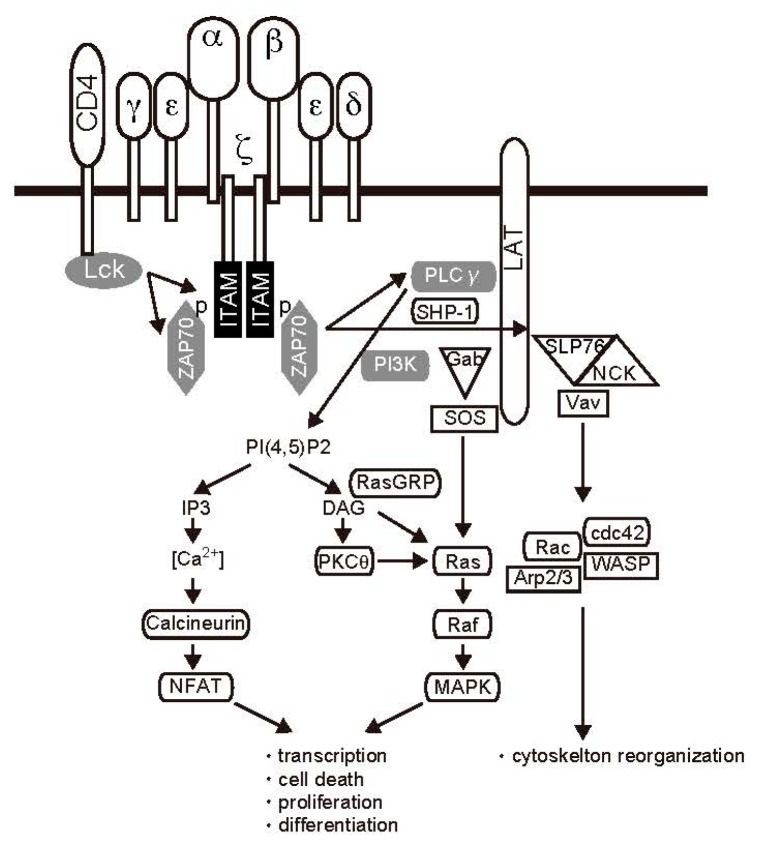
TCR signaling. Signaling is initiated by engagement of the T-cell receptor. Formation of the signaling complex coordinates numerous second messenger and kinase cascades, leading to transcriptional activation and cytoskeletal reorganization.

The genes encoding immunological mediators, such as cytokine IL-2, are transcribed in T cells after the TCR/CD3 complex and costimulatory molecules present on T cells are stimulated by the MHC/peptide complex on antigen-presenting cells (APCs) [17] (Figure 1). One of the first biochemical consequences of TCR binding is activation of Lck, a Src family protein tyrosine kinase. Lck bound to the cytoplasmic domains of CD4 and CD8 is activated in response to antigen presentation. Next, key substrates are phosphorylated, including specialized motifs in CD3 molecules [18]. Phosphorylation of immunoreceptor tyrosine-based activation motifs (ITAMs) creates docking sites for the Src-homology 2 (SH2) domains of ζ-chain-associated protein kinase 70 (ZAP70) [19]. As a result of ZAP70 recruitment, the TCR is effectively endowed with tyrosine kinase function, resulting in phosphorylation of phospholipase C (PLC) γ. PLCγ hydrolyzes the phospholipid phosphatidylinositol-4,5-diphosphate (PIP2) to form the second messengers inositol trisphosphate (IP3) and diacylglycerol (DAG) [5]. Production of DAG results in the activation of two major pathways involving rat sarcoma (Ras) and protein kinase C (PKC) θ. Ras is a guanine nucleotide-binding protein required for activation of the serine-threonine kinase Raf1, which initiates a mitogen-activated protein kinase (MAPK) phosphorylation and activation cascade. Raf1 is a MAPK kinase kinase, which phosphorylates and activates MAPK kinase (MAPKK). MAPKKs then phosphorylate and activate the MAPK extracellular signal-regulated kinase (ERK) [20], which contributes to the activation of the activator protein-1 (AP-1; Jun/Fos) transcription complex via regulation of target gene expression [21]. The PKCθ signaling pathway is also regulated by DAG and modulates nuclear factor-κB (NF-κB) activation. After stimulation with DAG, PKCθ undergoes conformational changes, and its activated form then phosphorylates caspase recruitment domain membrane-associated guanylate kinase protein 1 (CARMA1), leading to a conformational change that allows CARMA1 to associate with B-cell CLL/lymphoma 10 (Bcl-10) and mucosa-associated lymphoid tissue 1 (MALT1). This complex, which is called CBM, promotes IκB kinase (IKK) activation [22,23]. NF-κB is normally kept inactive in the cytoplasm by interaction with its inhibitor IκB. Activated IKK phosphorylates IκB at specific serine residues, leading to proteasomal degradation. Degradation of IκB releases NF-κB, which then migrates to the nucleus and regulates gene expression [24]. 

IP3 generated by PLC stimulates Ca^2+^-permeable ion channel receptors on the endoplasmic reticulum (ER) membrane, leading to the release of ER Ca^2+^ stores into the cytoplasm. Decreasing Ca^2+^ triggers a sustained influx of extracellular Ca^2+^ through the activation of plasma membrane Ca^2+^ release-activated Ca^2+^ (CRAC) channels in a process known as store-operated Ca^2+^ entry [25]. Stromal interaction molecule is the sensor of depleted ER Ca^2+^ stores and activator of CRAC channels [26,27]. TCR-induced increases in intracellular Ca^2+^ levels result in activation of the phosphatase calcineurin and Ca^2+^/calmodulin-dependent kinase [28], which in turn activates a variety of transcription factors [29]. Activated calcineurin dephosphorylates members of the NFAT family, leading to their translocation to the nucleus. Thus, several transcription factors, such as NF-κB, AP-1, and NFAT, play important roles in the response to TCR stimulation [30]. Cytokine production during this process has important implications for the elimination of foreign antigens.

### 2.2. Cytokine Receptor Signaling

T cells receive antigen stimuli via the TCR; however, they cannot effectively eliminate foreign antigens with signaling from TCR alone. Cytokines regulate the activity and/or amplitude of effector functions in T and other immune cells. Intercellular transmission of messages by cytokines is essential for immune regulation. Antigen and cytokine stimuli via the TCR and cytokine receptor induce different subsets of helper T cells [31]. IL-12 produced by APCs promotes the differentiation of naïve CD4 T cells into type 1 helper T cells (Th1 cells) to stimulate cellular immunity. Additionally, induction of Th2 and follicular T cells is essential for production of antigen-specific antibodies in humoral immunity. IL-4 and IL-21 stimulate the maturation of these helper T cells from naïve T cells [32]. Macrophage and dendritic cells function simultaneously as APCs and IL-12-producing cells to induce the development of Th1 cells. APCs also contribute to the expression of co-stimulatory molecules. Engagement of CD28 on T cells by CD80 or CD86 on APCs is required for T-cell priming. IL-12 from macrophage and dendritic cells is a potent and essential inducer of differentiation in interferon γ (IFNγ)-producing cells [33]. IL-12 production is clearly correlated with sensitization of Th1 lymphocytes [34]. CD8a^+^ dendritic cells from IL-12-deficient mice do not sensitize Th1 cells when injected into wild-type recipients, whereas the same subset of dendritic cells from IL-12-sufficient animals induces a polarized Th1 response [35]. This observation indicates that transferred macrophages or dendritic cells directly induce polarization of T cells. Adaptive transfer experiments suggest that CD8a^+^ dendritic cells are required for Th1 development in vivo. In contrast, the requirements of Th2 induction may be less stringent. Administration of macrophages or CD8a dendritic cells pulsed with antigen induces Th2 differentiation [33]. IL-4 secreted from APCs is essential for induction of Th2 cells from naïve T cells. Taken together, these observations strongly suggest that macrophages and dendritic cells play essential roles as APCs in the polarization of Th cells. 

Cytokines function by binding receptors on the surface of target cells. These receptors can be categorized into several groups according to their structures [36,37]. Type I cytokine receptors have four Cys residues and a Trp-Ser-X-Trp-Ser (WSXWS) sequence in their extracellular domains. This group includes receptors for many interleukins, such as IL-2 and IL-3, and for granulocyte-macrophage colony-stimulating factor (GM-CSF). Type II cytokine receptors, including IL-10, IFNα, INFβ, and IFNγ receptors, have conserved Cys residues in both the N- and C-termini of their extracellular domains. Type III cytokine receptors include the tumor necrosis factor (TNF) receptor super family, whereas the IL-1 receptor is a type IV receptors. Receptors for chemokines, such as IL-8, are grouped in the chemokine receptor family, containing G-protein-coupled receptors with seven transmembrane domains.

Most cytokine receptors are composed of subunits common to multiple receptor types; for example, IL-3, IL-5, and GM-CSF receptors all share the same β subunit [37]. The IL-6 receptor subunit gp130 is also utilized by the IL-11 and oncostatin M receptors, whereas IL-2 receptor subunit γ is shared by the IL-2, IL-4, and IL-7 receptors. This overlapping distribution of receptor subunits is considered indispensable for cytokine redundancy [38]. Another characteristic of cytokine receptors is that they may consist of a combination of subunits that show low-affinity and high-affinity ligand binding. For example, IL-3R is composed of two subunits, the α-chain and β-chain. The β-chain binds IL-3 specifically, but cannot bind IL-3 in the absence of the α-chain. In the presence of the α-chain, however, the β-chain forms high-affinity IL-3R, which is capable of signal transduction [37]. Another example is IL-2R, which in its high-affinity form consists of an α-chain, β-chain, and γ-chain. The IL-2R has different forms, all showing varying binding activities; for example, the form containing the α-chain only shows low affinity, the β-γ complex shows intermediate affinity, and the complex with all three subunits shows high affinity [39].

### 2.3. The Janus Kinase (JAK)/STAT Pathway

When a ligand binds the extracellular domain of a receptor, its subunits combine to activate an intracellular signaling cascade. Such signaling mechanisms have been analyzed in detail. Cytokine receptor family members do not possess intrinsic kinase activity [37]. After receptor activation, cytokine signals are transmitted into the cellular compartment by phosphorylation of signaling molecules, such as adaptor protein and downstream kinases. In addition, because kinase inhibitors block cytokine signal transduction, we expect that tyrosine kinases bind to these receptors. 

This hypothesis was proven by the discovery of the JAK family of tyrosine kinases [40,41]. Introduction of a DNA fragment encoding tyrosine kinase 2 (Tyk2), a JAK family protein, rescues IFNα reactivity in RLM-11-1 cells, which are mutant T cells unresponsive to IFNα. Four JAK family members have been identified in mammals: JAK1, JAK2, JAK3, and Tyk2 [42]. Proteins in this family have two kinase domains. Although JAK3 is restricted to the hematopoietic system, the other JAK family members are expressed in a variety of tissues. These kinases bind the Box1 and Box2 motifs of cytokine receptors present in their cytoplasmic juxtamembrane regions. Subunit assembly causes bound JAK molecules to phosphorylate one another at Tyr residues. This self-activation causes JAK to then phosphorylate Tyr residues in the cytoplasmic region of the receptor, acting as binding sites for downstream factors, including the STAT family of transcription factors. In the high-affinity IL-2R (i.e., the form composed of an α-, β-, and γ-chain), JAK3 phosphorylates Y392 and Y510 in the IL-2Rβ chain in an IL-2-dependent manner [39]. STAT5 then binds to these phosphorylated Tyr residues on the IL-2Rβ chain and is phosphorylated at Y694, conferring on it transcriptional activity. 

**Figure 2:**
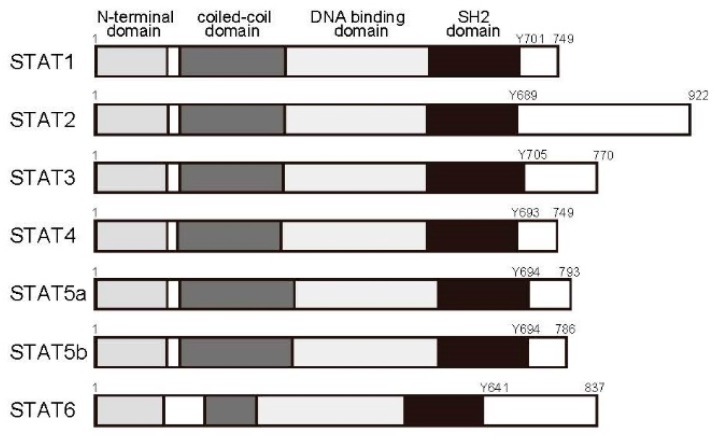
STAT protein structure. STAT proteins possess conserved domains, such as the N-terminal oligomerization domain (NTD), coiled-coil domain (CC), DNA-binding domain (DNBD), SH2 domain (SH2), and transactivation domain (TAD). Phosphorylation of tyrosine at the C-terminus is essential for STAT activation.

Seven STAT family proteins have been identified to date: STAT1, STAT2, STAT3, STAT4, STAT5a, STAT5b, and STAT6 (Figure 2). Each contains the SH2 domain and Tyr residues in the carboxyl terminus. STAT proteins reside in the cytoplasm in resting cells. Once a receptor reacts to a ligand, the ligand is recruited to the cytoplasmic domain of the receptor, where the carboxyl-terminal Tyr residues are phosphorylated by JAK. After phosphorylation, STATs bind the SH2 domains of other STAT5 proteins to form homodimers or heterodimers, which then leave the receptor and migrate to the nucleus to promote target gene expression. 

### 2.4. The Ras/MAPK Pathway

Activated JAK phosphorylates Shc, an adaptor protein that binds receptors [43,44,45]. Shc binds the Ras pathway protein son of sevenless (SOS) and growth factor receptor binding protein 2 [46]. SOS exchanges GDP for GTP on Ras to activate it, which then activates the downstream MAPK family proteins ERK1 and ERK2 [47]. MAPKs not only regulate STAT activity but also induce expression of the transcription factors c-Jun and c-Fos [44,48,49]. MAPKs can stabilize dimerized STAT molecules by additional phosphorylation at serine residues, further increasing the transcriptional activity of the STAT complex [50,51,52,53]. 

### 2.5. The Phosphatidylinositol 3-Kinase (PI3K)/Akt Pathway

Cytokine receptors, such as IL-2R and IL-3R, activate PI3K when stimulated by ligand binding [54]. This lipid kinase catalyzes the production of phosphatidylinositol (3,4,5)-trisphosphate (PI(3,4,5)P3), a key signaling molecule. PI3K is composed of a regulatory subunit (p85) and a catalytic subunit (p110). The p85 subunit binds to the Tyr-phosphorylated cytoplasmic domain of receptors subsequent to cytokine stimulation; consequently, the p110 subunit phosphorylates membrane phosphatidylinositol at carbon 3 of the inositol ring. p110 can be activated directly by Ras or, in some cases, by JAK [55]. Akt, which is involved in cell survival, proliferation, and motility, is activated downstream of PI3K.

Cytokines activate many other pathways in addition to those described herein; interested readers should consult other reviews for more details.

## 3. Signaling Regulation and Autoimmune Disease

Antigen-stimulated helper T cells differentiate into subsets of Th cells depending on the surrounding cytokines. Interest in how these signals are controlled has increased as our knowledge of the mechanisms involved has expanded, particularly after the observation that intracellular signal pathways show abnormal activation in many autoimmune diseases. 

### 3.1. Negative Regulation by Protein Tyrosine Phosphatases (PTPs)

PTPs regulate kinase activities by dephosphorylating tyrosine residues involved in catalytic function [56,57]. Several studies have demonstrated the roles of PTPs in the regulation of the JAK signaling pathway [58,59,60]. Src homology region 2 domain containing phosphpatase-1 (SHP-1) has been shown to inhibit JAKs following their recruitment to receptor complexes, which is facilitated by their binding to receptors via the SH2 domain of SHP-1. Moreover, SHP-1 has been shown to bind JAKs directly and mediate their dephosphorylation [50,60,61,62,63]. This phosphatase also inhibits the IL-4-mediated Th2 response [64] through blockade of both the JAK and MAPK signaling pathways. SHP-1 mediates inhibition through immune-receptor tyrosine-based inhibitory motif (ITIM)-containing receptors, such as the TCR, by dephosphorylating ZAP70 [65]. Additionally, SHP-2 has an important role in the negative regulation of JAK in IFN-stimulated cells [66,67]. Researchers have performed detailed characterizations of the specific mechanisms involving SHP-2 and JAK [68,69]. Importantly, the SHP-2/JAK association does not require the SH2 domain of SHP-2 or the kinase-like domain of JAKs. SHP-2 is tyrosine phosphorylated by JAK1 and JAK2, but not JAK3, on Y304 and Y327 via direct binding. Finally, regulation of JAK by SH-PTP1 has been shown to involve IFN signaling [50], and SH-PTP1 has been shown to be involved in the inactivation of JAK2 and the abrogation of proliferative signals [70].

### 3.2. JAK/STAT Pathway Regulation by the Cytokine-Induced SH2-Containing Protein (CIS)/Suppressor of Cytokine Signaling (SOCS) Family

**Figure 3:**
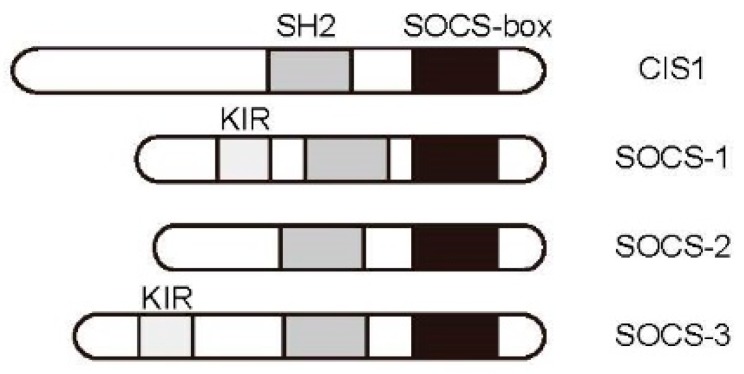
SOCS family proteins. SOCS members share a central SH2 domain and carboxyl terminal SOCS Box. SOCS1 and SOCS3 possess a kinase inhibitory region (KIR) that serves as a pseudo-substrate for JAKs.

The integrity of the JAK/STAT pathway is strictly maintained by several molecules. CIS/SOCS proteins are a group of negative regulators of cytokine signaling that play important roles in the maintenance of homeostasis, including the retraction of lymphocyte activity. This family was independently identified by several research groups. CIS was first identified [71], followed by SOCS-1 [72,73,74]. CIS/SOCS family proteins share a conserved structure consisting of an SH2 domain and a SOCS Box domain [75]. The family consists of eight members, of which CIS, SOCS-1, SOCS-2, and SOCS-3 are involved in regulating cytokine signaling (Figure 3).

The gene encoding CIS (Cish) was first cloned as an immediate early gene whose expression was shown to be upregulated following IL-2 or IL-3 stimulation. Subsequent analysis found that this factor bound directly to cytokine receptors [71]. Its overexpression inhibits cytokine-inducible cell proliferation. SOCS-1 was first identified as a protein that bound directly to JAK2 to inhibit kinase activity [76]. After binding of the SH2 domain and the activation loop of JAK2 at Y1007, the amino-terminal kinase inhibitory region (KIR) binds the active pocket, inhibiting substrate phosphorylation [75]. SOCS-3 was first identified as a molecule that inhibits JAK binding to cytokine receptors [77]. In contrast to SOCS-1, this protein binds to receptors specifically at phosphorylated Tyr residues in the cytoplasmic domain and is believed to inhibit nearby JAK proteins via the KIR motif. SOCS-1 is capable of blocking many cytokine signals, but is primarily induced by IFNγ and IL-4, suggesting that its activity is important in Th1 and Th2 cells [75].

Animal model-based studies have reported cytokine signaling regulation by the CIS/SOCS family to be involved in several autoimmune diseases. Activation of STAT1 and STAT3 due to increased production of inflammatory cytokines has been observed in inflammatory bowel disease, as have elevated Th1 cell counts [78]. STAT3 phosphorylation increases under severe disease conditions, whereas SOCS-3 expression is observed during patient recovery. Dextran sulfate sodium-induced colitis has been induced in transgenic mice expressing a point mutation in SOCS-1 (SOCS-1-F59D), which inhibits the activity of SOCS family proteins [78]. These mice show markedly worse intestinal inflammation than wild-type controls and exhibit associated weight loss. Thus, CIS/SOCS family proteins prevent intestinal inflammation. STAT5 activation and SOCS-3 have also been reported to be involved in collagen-induced arthritis (CIA) in a well-established mouse model of rheumatoid arthritis (RA) [79]. Self-reactive T cells are strongly implicated in these arthritis models because they are antigen-inducible. Adenoviral *SOCS-3* gene transfer significantly improved symptoms in a mouse model of CIA, and SOCS-3 has also been shown to have positive effects related to suppression of IL-6 production, a process closely connected to CIA pathology [80].

### 3.3. Regulation of TCR Signaling and Associated Diseases

Helper T cells are activated when TCRs on their surfaces recognize antigen peptides and MHC class II (MHC-II) molecules, activating associated CD4 coreceptors [5]. Once activated, Lck bound to the cytoplasmic domain of CD4 phosphorylates Tyr residues with an ITAM in nearby CD3 within the TCR complex [18]. This series of reactions triggers the recruitment of ZAP70, another tyrosine kinase, to the CD3 ITAM, thereby initiating TCR signaling. Evidence of T-cell infiltration in inflamed joints, associations of specific MHC-II haplotypes with disease sensitivity, and symptomatic improvement following T-cell depletion has suggested that T cells and TCR signaling may play a pivotal role in disease [81]. However, the relationship between TCR signaling and autoimmune disease remains unclear. 

This relationship has been studied in SKG mice, a mouse model that spontaneously develops chronic inflammatory arthritis resembling human RA [82]. In these mice, swelling in the finger joints began eight weeks after birth and progressed to chronicity, spreading to other joints in the fore- and hindpaws. Histopathological observations showed synovial cell proliferation and inflammatory cell infiltration in the inflamed joints. Other pathological changes in their joints included pannus formation and destruction of osteal tissue. In a search for the molecular cause of spontaneous arthritis in this mouse model, a point mutation in the SH2 domain of ZAP70, which altered codon 163 from tryptophan to cysteine (W163C), was identified. TCR signal strength is attenuated by the ZAP70^W163C^ mutation, resulting in abnormal T-cell maturation in the thymus [82]. Therefore, this point mutation alters the sensitivity of thymocyte development during thymic selection, preventing elimination of some with the self-reactive repertoire. 

### 3.4. T Cell-Targeted Nanomedicine

Leukemia inhibitory factor (LIF) is a pleiotropic cytokine of the four-α-helix bundle family that includes IL-6, LIF, oncostatin M, and IL-11 [83]. The LIF protein is a monomeric glycoprotein of 180 amino acid residues and includes a disulfide bound. The cytokine receptor gp130 is the shared signaling subunit of the IL-6 family of cytokines. The LIF receptor is composed of a gp130 and gp190 heterodimer [84], and LIF-mediated binding of the receptor activates several pathways, including the JAK/STAT, PI3K/Akt, and MAP kinase pathways [84,85]. LIF is essential to the survival of hematopoietic stem cells, and is released from T cells in response to activation [86]. In mice, isogenic clones of Th1, Th2, and Treg cells are the major sources of LIF [87]. Recently, it has been shown that activated human Treg cells also release high levels of LIF [88]. LIF supports expression of Foxp3 and is associated with Treg cell maintainence and immune tolerance. Therefore, LIF has been applied in anti-inflammatory strategies to control inflammation [89]. Anti-CD4 monoclonal antibody-coated PLG (poly(lactide-co-glycolide)) nanoparticles have been used to deliver LIF to CD4 T cells, promoting CD4^+^ CD25^+^ Foxp3^+^ Treg cell development [90,91]. Nanoparticle-mediated delivery was found to promote Treg cell expansion and control inflammation. Targeted nanoparticles provide a powerful new access rout to T-cell developmental plasticity in autoimmune diseases. 

## 4. T-Cell Signaling Inhibitors and Autoimmune Diseases

Self-reactivity is mediated by immune tolerance at the organismal level. The mechanisms inhibiting signaling pathways have also been evaluated at the cellular level. Disruption of endogenous regulatory pathways at both the cellular and organismal levels can lead to autoimmune disease. This section summarizes the molecular targeted agents used to control autoimmune diseases, focusing on examples of major drugs that have been analyzed in animal models of diseases or have already been approved for medical treatment. 

Lipid molecules present in the lipid bilayer of cells not only help to maintain separation between the interior of cells and the external environment, but also contribute to intracellular signaling. Phosphoinositides, a type of cellular membrane lipid, are phosphorylated by PI3Ks to produce phosphorylated inositol lipids [54]. This enzyme family is divided into three groups, namely, Class I, Class II, and Class III, of which Class I PI3Ks have been analyzed in detail. The Class I members PI3Kα, PI3Kβ, PI3Kδ, and PI3Kγ catalyze the conversion of phosphatidylinositol-4,5-bisphosphate to phosphatidylinositol-3,4,5-triphosphate. Through this enzymatic reaction, the PI3K family regulates cellular functions, such as proliferation, survival, apoptosis, and migration. PI3Kδ and PI3Kγ, which are expressed in leukocytes, are deeply involved in the control of the immune system.

PI3Ks can be activated in T cells by TCR or IL-2 stimulation. Mice deficient in p110γ, a subunit of PI3Kγ, show reduced TCR stimulation-induced proliferation and cytokine production [92]. Similarly, T cells derived from knockout mice with defective PI3Kδ respond weakly to TCR stimulation in vitro and show reduced antigen-specific responses in vivo [93]. p110δ mutant mice have reduced levels of regulatory T cells in secondary lymphoid organs, suggesting that PI3Kδ may be important for the maintenance of regulatory T cells in peripheral tissue [94]. Based on these findings and others, PI3Kγ and PI3Kδ have attracted attention as molecular targets for treating autoimmune disease [95,96]. Haruta et al. found that ZSTK474, a PI3K-specific inhibitor, suppresses the onset of adjuvant-induced arthritis (AIA) in a rat model [97]. In this animal model, T cells propagate in the lymph nodes before AIA onset. This proliferation was markedly suppressed in rats treated with ZSTK474. To measure direct inhibition of T cells, T cells prepared from rat lymph nodes were stimulated in vitro. Production of IL-17, a cytokine closely linked to arthritis, was significantly suppressed by ZSTK474. The results suggested that ZSTK474 inhibited T cells in the early stages of the disease, and it is therefore expected that PI3K-targeting compounds could prevent autoimmune disease. 

Similar to TCR stimulation, cytokine stimulation is closely linked to the pathogenicity of autoimmune diseases. Cytokines stimulate immune cells by binding receptors to activate intracellular signaling pathways. Phosphorylation of tyrosine residues is observed at the beginning of this process, which is catalyzed by JAK bound to the intracellular domains of cytokine receptors. Thus, the JAK family is targeted to regulate excessive cytokine levels as a strategy for controlling disease. Organisms are equipped with endogenous control mechanisms to inhibit JAK/STAT signaling molecules, such as the SOCS family; however, these regulatory pathways lose their effectiveness in patients with autoimmune diseases. Several JAK inhibitors have been reported in recent years.

Tofacitinib was developed as a JAK inhibitor capable of suppressing IL-2-inducible T-cell proliferation [98]. This compound can inhibit JAK1, JAK2, and JAK3. Its therapeutic efficacy has been investigated in a CIA mouse model based on its hypothesized efficacy in treating RA induced by autoreactive T cells [99]. CIA mice treated with tofacitinib via an osmotic pump exhibited improvements in joint symptoms in a dose-dependent manner [98]. Their reductions in clinical scores were equivalent to those in the control group treated with anti-TNF-α antibodies. Furthermore, pathological changes to joint tissue and serum levels of inflammatory cytokines were both reduced in the treatment group, as was expected from the improvement of clinical signs. Comparable clinical improvements were observed in parallel experiments using an AIA rat model, raising expectations for this compound’s usefulness as a molecular targeting agent. In general, Th1 and Th17 cells are activated and play pathogenic roles in RA. However, when animals are treated with tofacitinib, it is unclear which cells are targeted to prevent disease symptoms. Tofacitinib inhibits IFNγ and IL-17 production from activated CD4^+^ cells [100]. Based on the activity of tofacitinib, the inhibitory mechanism that prevents functional maturation from naïve to effector T cells was analyzed. Tofacitinib inhibits CD4 T-cell maturation into Th1 and Th17 cells [101]. Despite its demonstrated efficacy in treating CIA, tofacitinib has also been found to promote Th17 differentiation and exacerbate disease severity in an experimental autoimmune encephalomyelitis system, as shown in an animal model of multiple sclerosis in humans, when administered at low doses [102]. Since then, tofacitinib has been evaluated in several clinical trials and was approved for use as an anti-rheumatic drug [102,103,104,105,106,107]. 

Other JAK inhibitors have been developed as well; clinical trials and approval for the use of baricitinib followed soon after those for tofacitinib [108,109,110,111]. Recently, a novel chemically synthesized JAK inhibitor, ASP015K (peficitinib), was developed [112]. This compound blocks JAK1 and JAK3 with 50% inhibitory concentrations of 3.9 and 0.7 nM, respectively. ASP015K prevented IL-2-induced STAT5 phosphorylation. In addition, ASP015K dose-dependently improved paw swelling in a rat adjuvant-induced arthritis model. Therefore, this compound may have therapeutic potential for the treatment of RA. Further investigations to clarify this potential are warranted. 

Tumor necrosis factor-α (TNFα) plays an important role in the pathogenesis of inflammatory disorders, such as RA. Two receptors of TNFα (TNFR) have been identified: TNFR1 and TNFR2 [113,114]. These receptors have the same extracellular domains with one major difference, the presence of a cytoplasmic death domain (DD) on TNFR1 and its absence on TNFR2 [115]. Upon binding of TNFα to TNFR, signaling is initiated through association of the TNFα DD to the TNFR-associated death domain (TRADD). Three protein adaptors then bind to TRADD: receptor-interacting protein 1 (RIP1), TNFR-associated factor 2 (TRAF2), and Fas-associated DD (FADD). TNFR1 stimulates cell survival or activation, and TRAF2 is recruited. TRAF2 then stimulates two major signaling pathways: the nuclear transcription factor (NF)-κB and AP-1 pathways [116]. Once the NF-κB essential modulator (NEMO) complex, which comprises IKKα, IKKβ, and IKKγ, is activated by TRAF2 [116], it causes phosphorylation of the NF-κB inhibitor IκB, which leads to NF-κB translocation into the nucleus to induce cytokine gene transcription [117,118]. MAPK is also activated by TRAF2 to promote c-Jun NH2-terminal kinase intern activation that in turn phosphorylates c-Jun, increasing its activity [119]. Excessive TNFα can trigger production of diverse pro-inflammatory cytokines, such as interferon-γ, IL-1, IL-6, IL-8, and inflammatory markers. This suggests that TNFα is located upstream of the cytokine cascade, and its inhibition may be useful for management of inflammatory arthritis [120,121].

Several strategies have been adapted to produce biological agents capable of inhibiting TNFα. One of major category of such agents is monoclonal antibodies against TNFα [122]. Infliximab [123] and adalimumab [124] neutralize the activity of TNFα. These monoclonal antibodies bind membrane-bound TNFα on the cell surface, fixing complements and inducing cell lysis, thereby inhibiting TNFα production. Therefore, TNFα inhibitors prevent TNFα-induced inflammatory cascades and disease progression. In fact, the usefulness of antibodies in combination with methotrexate or other biological agents clearly lies not only in the induction of clinical remission but also in the arrest of damage in RA [125]. Because TNFα blocks infectious diseases caused by intracellular pathogens, such as mycobacteria, *Pneumocystis carinii*, and other fungi [126,127,128,129], and also induces tumor cell lysis, its inhibition may result in increased incidence of opportunistic infections and malignancy of cancers. Therefore, development of molecular targeted agents is necessary for safe control of autoimmune diseases.

Many inhibitors targeting single protein kinases are in clinical trials. Such molecular targeted agents block enzymes specifically and improve clinical outcomes. However, adverse events, such as elevations of serum creatinine, decreases in neutrophil count, and infection, are also reported. Although the majority of adverse events are manageable, opportunistic infections, such as pulmonary tuberculosis and pneumocystis pneumonia, have been reported [130]. Thus, although some therapies have been approved in United States, Japan, and other countries, careful surveillance for infection and malignancy and the accumulation of long-term safety data are required. 

## 5. T-Cell Function and Cytoplasmic Acetylation

Post-translational modification is closely linked to protein function. Recent studies have demonstrated that lysine acetylation regulates protein activity [9,10,131,132]. Acetylation is a post-translational modification that occurs in the nucleus, similar to omnipresent histone modifications, but is also known to support cellular functions in the cytoplasm [133,134]. However, the importance of cytoplasmic acetylation remains unclear. Microtubules are a type of protein known to be acetylated in the cytosol, where they are involved in the control of cell structure. Additionally, recent studies have reported cytoplasmic acetylation of receptors and signaling molecules [9,11,131].

### 5.1. Microtubule Regulation by Cytoplasmic Acetylation and T-Cell Function

Microtubules are structures indispensable to forming and maintaining cell shape and provide scaffolding for transporting materials and arranging organelles within the cytoplasm [135,136]. Microtubules are polar structures with “plus” and “minus” ends; the direction of any transport depends on this polarity. In order for cells to form their complex morphological shapes, microtubules must be oriented properly. In motile cells, microtubules are oriented with the plus end on the extending, leading edge [137]. During cell division, microtubules form spindles by extending, plus-end first, from the two spindle poles to connect to kinetochores present on the chromosome. Microtubule orientation varies dynamically in space and time, and the molecular mechanisms that control this behavior have yet to be elucidated.

Microtubules are cylindrical structures formed by the polymerization of heterodimers of α-tubulin and β-tubulin. Their plus ends exhibit stochastic competition between polymerization and depolymerization, making them dynamically unstable. This instability is regulated by various proteins, but also controlled by post-translational modifications, including acetylation [138]. Acetylation of α-tubulin causes it to form heterodimers with β-tubulin, which then combine to form filaments, whereas deacetylation of α-tubulin triggers its dissociation from heterodimers, leading to depolymerization. Acetylation control seems to play a role in the stabilization of microtubule structures, and accordingly, in the regulation of T-cell migration. The function of acetylation is not limited to structural modulation; indeed, acetylation has been found to alter the binding specificities and activities of microtubule-binding proteins as well as a variety of motor proteins, which allow establishment of polarity and control of transport inside the cell. Immune synapses (ISs) form at the interface between a T cell and an APC during antigen recognition. Mitochondria are transported to the ISs and ferried along microtubules by motor proteins, replenishing ATP consumed on the cytoplasmic side of the complex [139]. These organelles clustered at the IS contact zone also help regulate intracellular Ca^2+^ levels. Acetylation and deacetylation of tubulin also regulate the disorganization of signaling milecules at the IS and diminished production of IL-2 [140]. TCR-related molecules, such as CD3 and ZAP70, move along microtubules in a dynein-dependent manner [141]. Suppression of dynein perturbs IS formation. These results suggest that microtubule acetylation in the cytoplasm regulates the T-cell response.

### 5.2. Regulation of Cytokine Cascades by Acetylation of Signaling Molecules in the Cytoplasm

Antigen-specific T cells propagate after interaction with their cognate antigens. Stimulation of IL-2R and TCR complexes plays an important role in the T-cell proliferation process. High-affinity IL-2R consists of three subunits, i.e., an α-chain, β-chain, and a γ-chain, each of which contains an IL-2-binding domain on the extracellular region of the receptor [39]. The α-chain does not participate in signal transduction, because its cytoplasmic domain consists of only 13 amino acid residues. The cytoplasmic domain of the IL-2R β-chain contains 286 residues, which can be divided into (from membrane-proximal to -distal) a Box1 motif, a serine-rich (S) region, an acidic (A) region, and a proline-rich (P) region. The γ-chain contains 86 residues. Two Src homology domains are present. IL-2R signaling is carried out by the β- and γ-chains. JAK1 binds to the S region of the β-chain, whereas JAK3 binds to a C-terminal 48-residue sequence in the γ-chain; they are activated when IL-2 binding brings the two subunits into close proximity. The activated JAK proteins phosphorylate tyrosine residues within the β-chain, including Y338 in the A region, which then acts as a binding site for the adaptor protein Shc, triggering the MAPK and PI3K pathways [39,47]. STAT5 binds to phosphorylated Y392 and Y510 in the β-chain and is phosphorylated by JAKs. Subsequently, phosphorylated STAT5 forms homodimers and migrates to the nucleus to promote the transcription of target genes, such as *IL-2* and *CIS* [71]. 

**Figure 4:**
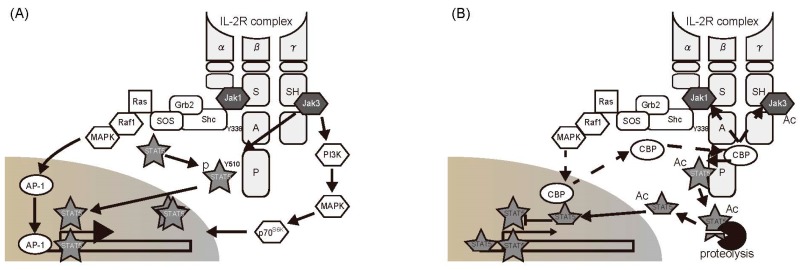
Schematic of IL-2 receptor signaling pathway: (**A**) An IL-2 signal presumably involves the integration of these and other pathway as well. (**B**) CBP-mediated acetylation of STAT5 negatively regulates IL-2 receptor signaling.

In essence, the status of a T cell depends on the state of its intracellular signaling pathways. T-cell activities are suppressed after elimination of their target pathogens. TCR- and IL-2R-mediated signals are inhibited by phosphatases and the SOCS system, as discussed previously. In a recent study, we found that cytoplasmic acetylation plays a role in the negative control of IL-2R signaling via the JAK/STAT5 pathway [9]. The murine T-cell line CTLL-2 undergoes proliferation in response to JAK/STAT5 activation following IL-2 stimulation. STAT5 was immunoprecipitated from cell extracts prepared from CTLL-2 cells following IL-2 stimulation. The isolated STAT5 exhibited both phosphorylation and acetylation [9]. The acetylated forms of JAK1 and JAK3 were also detected in this fraction. These findings suggested that an acetyltransferase binds to the IL-2R complex. Moreover, IL-2-dependent acetyltransferase activity was detected in anti-IL-2Rβ-chain fractions. This fraction was then subjected to mass spectrometer analysis, and cyclic AMP responsive element binding protein (CREB)-binding protein (CBP), which catalyzes histone acetylation, was identified. Further examination showed that, while CBP is located in the nucleus in resting cells, it is transported into the cytoplasm in response to IL-2 stimulation, where it binds to the P domain of the IL-2Rβ-chain [9] (Figure 4).

IL-2R signaling pathways can be analyzed using mutant cells lacking different domains of the IL-2Rβ-chain. IL-2R-mediated pathways inducing translocation of CBP from the nucleus to cytoplasm were investigated. Analysis using BAF-B03 cells showed defects in the IL-2R β-chain, resulting in expression of mutant IL-2R β-chains lacking the S, A, and P regions. The signal from the A region was essential for CBP migration from the nucleus. The A region signals the Lck, PI3K, and Shc/Ras pathways. Of these, the activity of Shc depends on phosphorylation at Y338. IL-2-dependent CBP transport was completely abrogated by BAF-B03 cells expressing a β-chain mutant in which Y338 was replaced by phenylalanine (Y338F). Moreover, experiments using inhibitors showed that the Shc-mediated RAS/MAPK pathway is involved in CBP transport, and that Lck is not involved. CBP-mediated acetylation was found to negatively regulate STAT5 transcriptional activity based on reporter assays and expression analysis of endogenous target genes. This mechanism may operate at the organismal level (physiological level) as well, since JAK/STAT5 inhibition by cytoplasmic acetylation was also observed in primary CD4^+^ T cells prepared from mice.

Other studies have reported that cytoplasmic acetylation occurs downstream of other receptors as well, including type I IFN, prolactin, and IL-7R [10,131,142]. Thus, the regulatory pathways involving acetylation are being elucidated. The role of this post-translational modification in regulating T-cell function via the IL-2 signaling pathway has already been demonstrated. Moreover, the JAK/STAT pathway is the target of acetylation in all cases reported to date. Many molecular targeting agents that act via the JAK/STAT pathway have already been well studied. A detailed analysis of the regulatory mechanisms mediating acetylation of components of the JAK/STAT pathway could facilitate advancements in our knowledge of the regulation of T-cell function and the control of autoimmune disease.

Even when symptoms appear similar, autoimmune diseases are often caused by different defects in the immune system. Thus, development of therapeutic approaches from many different angles is highly desirable. 

## 6. Conclusions

In this review, we discussed the regulatory mechanisms of signal transduction and T cell-mediated autoimmune diseases. Disruption of tissue function and integrity in immune diseases cannot be explained by T-cell responses alone. Nonetheless, some studies have shown that symptomatic relief from autoimmune diseases can be achieved through the regulation of T-cell signaling pathways, both by exogenous drugs and intrinsic mechanisms. Knowledge of the association between T-cell activity and signaling could serve as a basis for the therapeutic management of autoimmune diseases. In addition to the traditional role of phosphorylation, acetylation-mediated regulation of T-cell responses was also discussed. Improving our understanding of acetylation switches in T cells will promote the development of novel measures for disease control.

## Abbreviations

APCs	antigen-presenting cells
CD	cluster of differentiation
CIS	cytokine-induced SH2-containing protein
CREB	cyclic AMPresponsive element binding protein
DAG	diacylglycerol
ERK	extracellular signal-regulated kinase
IFN	interferon
IL	interleukin
IP3	inositol 1,4,5-triphosphat
ITAM	immune-receptor tyrosine-based activation motif
ITIM	immuno-receptor tyrosine-based inhibitory motif
JAK	Janus kinase
Lck	lymphocyte-specific protein tyrosine kinase
MAPK	mitogen-activated protein kinase
MEK	mitogen-activated protein/extracellular signal-regulated kinase
MHC	major histocompatibility complex
NEMO	NF-κB essential modulator
NFAT	nuclear factor of activated T cells
NF-κB	nuclear factor-κB
PIP2	phosphatidylinositol-4,5-diphosphate
PI3K	phosphatidylinositol 3-kinase
PKCθ	protein kinase Cθ
PLCγ	phospholipase Cγ
PTP	protein tyrosine phosphatase
RA	rheumatoid arthritis
Ras	rat sarcoma
SOCS	suppressor of cytokine signaling
Stat	signal transducers and activators of transcription
TCR	T-cell receptor
ZAP70	ζ chain-associated 70 kDa tyrosine phosphoprotein
